# The cuticular wax inhibitor locus *Iw2* in wild diploid wheat *Aegilops tauschii*: phenotypic survey, genetic analysis, and implications for the evolution of common wheat

**DOI:** 10.1186/s12870-014-0246-y

**Published:** 2014-09-16

**Authors:** Ryo Nishijima, Julio C M Iehisa, Yoshihiro Matsuoka, Shigeo Takumi

**Affiliations:** Graduate School of Agricultural Science, Kobe University, Rokkodai 1-1, Nada, Kobe, 657-8501 Japan; Department of Bioscience, Fukui Prefectural University, Matsuoka, Eiheiji, Yoshida, Fukui 910-1195 Japan

**Keywords:** Allopolyploid speciation, Cuticluar wax inhibitor, Synthetic wheat, Wheat evolution

## Abstract

**Background:**

Cuticular wax production on plant surfaces confers a glaucous appearance and plays important roles in plant stress tolerance. Most common wheat cultivars, which are hexaploid, and most tetraploid wheat cultivars are glaucous; in contrast, a wild wheat progenitor, *Aegilops tauschii*, can be glaucous or non-glaucous. A dominant non-glaucous allele, *Iw2*, resides on the short arm of chromosome 2D, which was inherited from *Ae. tauschii* through polyploidization. *Iw2* is one of the major causal genes related to variation in glaucousness among hexaploid wheat. Detailed genetic and phylogeographic knowledge of the *Iw2* locus in *Ae. tauschii* may provide important information and lead to a better understanding of the evolution of common wheat.

**Results:**

Glaucous *Ae. tauschii* accessions were collected from a broad area ranging from Armenia to the southwestern coastal part of the Caspian Sea. Linkage analyses with five mapping populations showed that the glaucous versus non-glaucous difference was mainly controlled by the *Iw2* locus in *Ae. tauschii*. Comparative genomic analysis of barley and *Ae. tauschii* was then used to develop molecular markers tightly linked with *Ae. tauschii Iw2*. Chromosomal synteny around the orthologous *Iw2* regions indicated that some chromosomal rearrangement had occurred during the genetic divergence leading to *Ae. tauschii*, barley, and *Brachypodium*. Genetic associations between specific *Iw2*-linked markers and respective glaucous phenotypes in *Ae. tauschii* indicated that at least two non-glaucous accessions might carry other glaucousness-determining loci outside of the *Iw2* locus.

**Conclusion:**

Allelic differences at the *Iw2* locus were the main contributors to the phenotypic difference between the glaucous and non-glaucous accessions of *Ae. tauschii*. Our results supported the previous assumption that the D-genome donor of common wheat could have been any *Ae. tauschii* variant that carried the recessive *iw2* allele.

**Electronic supplementary material:**

The online version of this article (doi:10.1186/s12870-014-0246-y) contains supplementary material, which is available to authorized users.

## Background

Cuticular wax production on aerial surfaces of plants has important roles in various physiological functions and developmental events; the wax prevents non-stomatal water loss, inhibits organ fusion during development, protects from UV radiation damage, and imposes a physical barrier against pathogenic infection [1-4]. The trait, the coating of leaf and stem surfaces with a waxy whitish substance, is called glaucousness. In common wheat (*Triticum aestivum* L., 2n = 6x = 42, genome constitution BBAADD), dominant alleles *W1* and *W2*, control the wax production and have been assigned to chromosomes 2B and 2D, respectively [5,6]. Additionally, dominant homoeoalleles for non-glaucousness, *Iw1* and *Iw2*, have also been mapped to the short arms of chromosomes 2B and 2D, respectively [6-9]. Wheat plants with either the *w1, w2*, *Iw1* or *Iw2* allele show the non-glaucous phenotype, indicating that *W1* and *W2* are functionally redundant for the glaucous phenotype and that a single *Iw* dominant allele is sufficient to inhibit the glaucous phenotype even in the presence of a *W1* or *W2* allele [3,6]. Wax composition in wheat plants with one *Iw* dominant allele is biochemically different from that in glaucous plants of any genotype; ß-diketones are completely absent from extracts of cuticular wax from *Iw* plants, while aldehydes and primary alcohols are very abundant in these extracts [3,10]. A fine map around the *Iw1* region on 2BS was constructed using an F_2_ population of tetraploid wheat (*Triticum turgidum* L., 2n = 4x =28, BBAA), and three markers tightly linked to *Iw1* were developed [10,11]. A high-resolution map of *Iw2* on 2DS has been developed in hexaploid wheat, and two markers tightly linked to *Iw2* were also developed [11]. Comparative mapping of *Iw1* and *Iw2* shows that the two loci are homoeologous to each other and orthologous to the same chromosomal region of *Brachypodium distachyon* (L.) P. Beauv. [11]. Recently, a third wax-inhibitor locus *Iw3* was identified on chromosome 1BS from wild emmer wheat [12], and a fine map of the *Iw3* locus is available [13]. *Iw2* is located on 2DS in *Aegilops tauschii* Coss. (2n = 2x = 14, DD), which is diploid and the progenitor of the D-genome of common wheat [14], but to our knowledge, a high-resolution genetic map of the *Iw2* region in *Ae. tauschii* has not been constructed.

Common wheat is an allohexaploid species derived from interspecific hybridization between tetraploid wheat with a BBAA genome and *Ae. tauschii*. Most cultivated varieties of tetraploid wheat are glaucous, even though non-glaucous types are frequently found among wild tetraploid accessions [6,15]; this variation indicates that the glaucous phenotype might have been a target of artificial selection during the domestication of tetraploid wheat. Glaucous accessions of *Ae. tauschii* are found in the area ranging from Transcaucasia to the southern coastal region of the Caspian Sea [5,16]. Almost all varieties of common wheat carry *W1* and *W2* and lack *Iw1* and *Iw2*; therefore, the D-genome donor of common wheat is assumed to have had the recessive *iw2* allele [5]. Glaucous *Ae. tauschii* accessions have the *W2* and *iw2* alleles. Non-glaucous accessions of *Ae. tauschii* that have the *W2* and *Iw2* alleles have been recovered from a wide distribution range in central Eurasia [5]. Moreover, discovery of a non-glaucous *Ae. tauschii* accession with the *w2* recessive allele has not yet been reported.

Therefore, analysis of the *Iw2* locus may provide important information that improves our understanding of the evolution of common wheat. Population structure analyses of *Ae. tauschii* indicate that the whole species *Ae. tauschii* can be divided into three major genealogical lineages, *tauschii* lineage 1 (TauL1), TauL2, and TauL3, and that genetically genomes of TauL2 accessions are most closely related to the D genome of common wheat [17-19]. Recently, a whole-genome shotgun strategy was used to generate a draft genome sequence of *Ae. tauschii* that has been published; this draft anchors 1.72 Gb of the 4.36 Gb genome to chromosomes [20]. A physical map of the *Ae. tauschii* genome that covers 4 Gb is also available [21]. The objectives of this study were (1) to examine the natural variation in glaucousness among a species-wide set of *Ae. tauschii* accessions, (2) to use F_2_ populations of *Ae. tauschii* accessions and synthetic hexaploid wheat lines to fine-map *Iw2* locus on 2DS, (3) to develop molecular markers that are closely linked to *Iw2* based on chromosomal synteny between barley and wheat chromosomes, and (4) to provide novel insights into the evolutionary relationship between the *Ae. tauschii* genome and the D genome of common wheat on the basis of the detailed genetic and phylogeographic knowledge of the *Iw2* chromosomal region.

## Methods

### Plant materials and phenotype evaluation

In all, 210 *Ae. tauschii* accessions were used in this study [22]. Their passport data, including geographical coordinates, have been provided in previous reports [23,24]. Previously, 206 of the *Ae. tasuchii* accessions were grouped into the three lineages, TauL1, TauL2, and TauL3, based on DArT marker genotyping analysis [19]. Of the 210 accessions, 12 were previously identified as subspecies *strangulata* based on the sensu-strico criteria [25,26]. Seeds from two *Ae. tauschii* hybrid F_2_ populations (n = 116 from each population) were sown in November 2011; one F_2_ population resulted from a cross between KU-2154 (non-glaucous) and KU-2126 (glaucous), the other from a KU-2003 (non-glaucous) by KU-2124 (glaucous) cross. In the 2012–2013 season, 169 additional F_2_ individuals of the KU-2154/KU-2126 population were grown to increase the size of the mapping population.

Previously, 82 synthetic hexaploid wheat lines were produced from crosses between a tetraploid wheat (*T. turgidum* subspecies *durum* (Desf.) Husn.) cultivar Langdon (Ldn) and 69 *Ae. tauschii* accessions [26,27]. These synthetic hexaploid wheat lines were used for crossing and phenotypic studies conducted in a glasshouse at Kobe University. Ldn shows the glaucous phenotype and is homozygous for the *iw1* allele [10]. Each synthetic hexaploid thus contained the A and B genomes from Ldn and one of many diverse D genomes originating from the *Ae. tauschii* pollen parents. In the present study, four F_3_ plants derived from one F_2_ plant of each synthetic hexaploid were grown individually during the 2007–2008 season in pots that were arranged randomly in the glasshouse; these 276 F_3_ plants were used for crossing and phenotypic observation. The following three pairs of synthetic hexaploids were used to generate three F_2_ mapping populations: Ldn/PI476874 (non-glaucous) and Ldn/KU-2069 (glaucous), Ldn/IG126387 (non-glaucous) and Ldn/KU-2159 (glaucous), and Ldn/KU-2124 (glaucous) and Ldn/IG47259 (non-glaucous). The first population (Ldn/PI476874//Ldn/KU-2069) comprised 106 F_2_ individuals grown in the glasshouse during the 2009–2010 season. Seeds from the other two populations were sown in November 2011, with the numbers of individuals in each being 100 (Ldn/KU-2159//Ldn/IG126387) and 82 (Ldn/KU-2124//Ldn/IG47259).

For analysis of the D genome of common wheat, 17 landraces collected in Iran were supplied from the National BioResource Project (NBRP) KOMUGI (http://www.shigen.nig.ac.jp/wheat/komugi). These Iranian landraces—KU-3097, KU-3098, KU-3121, KU-3126, KU-3136, KU-3162, KU-3184, KU-3189, KU-3202, KU-3232, KU-3236, KU-3274, KU-3289, KU-10393, KU-10439, KU-10480, and KU-10510—each showed the glaucous phenotype.

Glaucousness was evaluated based on the presence or absence of wax production on the surface of peduncles and spikes in both *Ae. tauschii* and synthetics. Wax production was clearly visible and whitish.

### Genotyping and construction of linkage maps

To amplify PCR fragments containing molecular markers, some of which were simple sequence repeats (SSRs), total DNA was extracted from leaves of the parental strains and F_2_ individuals. For SSR genotyping, 40 cycles of PCR were performed using 2x Quick Taq HS DyeMix (TOYOBO, Osaka, Japan) and the following conditions: 10 s at 94°C, 30 s at the appropriate annealing temperature (72, 73, or 75°C), and 30 s at 68°C. The last step was a 1-min incubation at 68°C. Information on SSR markers and the respective annealing temperatures was obtained from the NBRP KOMUGI web site (http://www.shigen.nig.ac.jp/wheat/komugi/strains/aboutNbrpMarker.jsp) and the GrainGenes web site (http://wheat.pw.usda.gov/GG2/maps.shtml). PCR products were resolved in 2% agarose or 13% nondenaturing polyacrylamide gels and visualized under UV light after staining with ethidium bromide. The MAPMAKER/EXP version 3.0b package was used for genetic mapping [28]. The threshold for log-likelihood scores was set at 3.0, and genetic distances were calculated with the Kosambi function [29].

Each polymorphism at the *Ppd-D1* locus on 2DS was detected with allele-specific primers and methodology described by Beales et al. [30]. A common forward primer, Ppd-D1_F (5′-ACGCCTCCCACTACACTG-3′), and two reverse primers, Ppd-D1_R1 (5′-GTTGGTTCAAACAGAGAGC-3′) and Ppd-D1_R2 (5′-CACTGGTGGTAGCTGAGATT-3′), were used for this PCR analysis. PCR products amplified with Ppd-D1_F and Ppd-D1_R2 detected a 2,089-bp deletion in the 5′ upstream region of *Ppd-D1* that is indicative of the photoperiod-insensitive *Ppd-D1a* allele [30]. EST-derived sequence-tagged site (STS) markers on 2DS, TE6, and WE6 were also used for genotyping; two STS markers for each locus, and these markers were previously developed along with the *Iw2*-linked markers [7]. The amplified PCR products were separated via electrophoresis through a 2% agarose or 13% nondenaturing polyacrylamide gel and then stained with ethidium bromide.

### Development of additional markers linked to *Iw2*

In our previous studies, we conducted deep-sequencing analyses of the leaf and spike transcriptomes of two *Ae. tauschii* accessions that represented two major lineages, and discovered more than 16,000 high-confidence single nucleotide polymorphisms (SNPs) in 5,808 contigs [31,32]. Contigs with the SNPs were searched with blastn against *Ae. tauschii* genome sequences [20] and barley genome sequences [33]; these genome sequences included high-confidence genes with an *E*-value threshold of 10^−5^ and hit length ≥ 50 bp, fingerprinted contigs, and whole genome shotgun assemblies.

To choose scaffolds for *Ae. tauschii* sequences throughout the *Iw2* chromosomal region, all the genes contained in each scaffold were searched with blastn against the barley genomic sequence using parameters described above. Scaffolds containing at least one gene aligned on the distal region of chromosome 2HS (between 3.66 Mb and 5.51 Mb) were considered possible candidates for marker development. Scaffolds without genes were anchored based on respective results from the blastn searches against the barley genome. First, high-confidence SNPs [31,32] plotted in this 2HS chromosomal segment were used for marker development to refine the target region. Next, SciRoKo version 3.4 [34] was used with search mode setting “mismatched; fixed penalty” to identify additional SSR markers in sequence data of candidate scaffolds. Additional SNPs were also identified on candidate scaffolds by sequencing approximately 700 bp of amplified DNA of two *Ae. tauschii* accessions, KU-2154 and KU-2126. The nucleotide sequences were determined using an Applied Biosystems 3730*xl* DNA Analyzer (Applied Biosystems, Foster City, CA, USA), and SNPs were found via sequence alignments constructed and searched with GENETYX-MAC version 12.00 software (Whitehead Institute for Biomedical Research, Cambridge, MA, USA).

For genotyping, total DNA was extracted from leaves taken from each of the 210 *Ae. tauschii* accessions and the 17 Iranian wheat landraces. SSR amplification and detection of polymorphisms at these loci were conducted as described above. The identified SNPs were then further developed into cleaved amplified polymorphic sequence (CAPS) or high resolution melting (HRM) markers. The primer sequences for each SNP marker and any relevant restriction enzymes are summarized in Additional file 1. PCR and subsequent analyses were performed as described previously [31,32,35].

### Blast analysis of the *Ae. tauschii* genes relative to the *Brachypodium* genome

Nucleotide sequences and annotation information of the selected *Ae. tauschii* scaffolds were analyzed with reference to the *Ae. tauschii* draft genome data, which was published by Jia et al. [20]. Reference sequences from *Brachypodium* [36] were searched against the National Center for Biotechnology Information (NCBI) NR protein database using the blastx algorithm with an *E*-value cut-off of 10^−3^.

### Association analysis of the linked markers with glaucousness

The Q + K method was conducted using a mixed linear model (MLM) function in TASSEL ver 4.0 software [37] for an association analysis by incorporating phenotypic and genotypic data and information on population structure. In a previous report, the Bayesian clustering approach implemented in the software program STRUCTURE 2.3 [38] was used with the setting *k* = 2 to predict the population structure of the *Ae. tauschii* accessions [19]. The Q-matrix of population membership probabilities was served as covariates in MLM. Kinship (K) was calculated in TASSEL based on the genotyping information of the 169 DArT markers for the 206 *Ae. tauschii* accessions [19]. We performed the *F*-statistics and calculated the *P*-values for the *F*-test, and the threshold value was set as 1E-3 for the significant association. We omitted the target markers from the association analysis when their minor allele frequencies were less than 0.05.

## Results

### Wax production variation among *Ae. tauschii* accessions and among synthetic wheat lines

**Figure 1:**
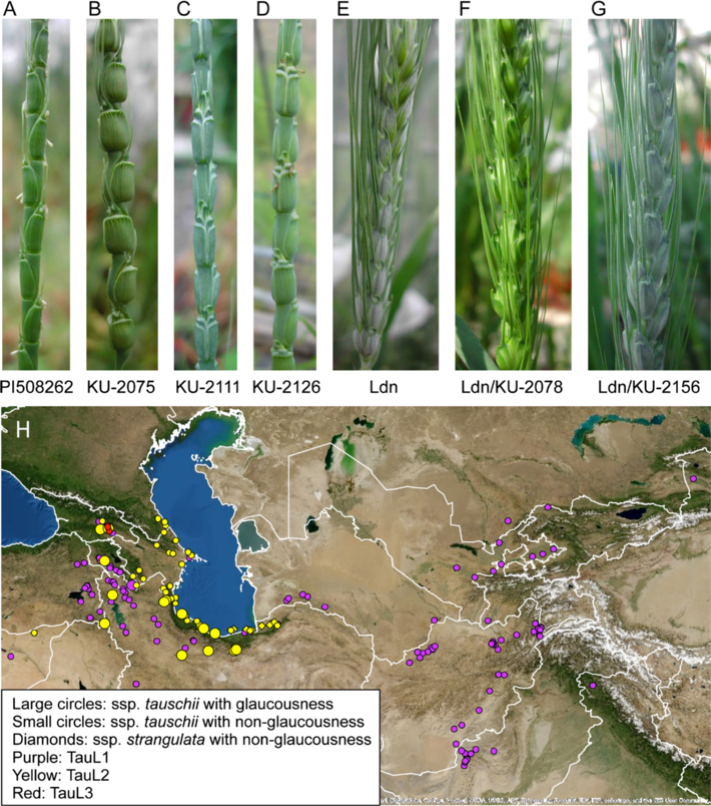
**Variation in cuticular wax production among** ***Ae. tauschii*** **accessions. (A,B)** Non-glaucous accessions of *Ae. tauschii*. PI508262 and KU-2075 are classified as subspecies *tauschii* and subspecies *strangulata*, respectively. **(C,D)** Glaucous accessions of *Ae. tauschii*. **(E)** A tetraploid wheat cultivar Langdon. **(F)** A synthetic hexaploid wheat line with the non-glaucous phenotype: the line was derived from an interspecific cross between Langdon and a non-glaucous *Ae. tauschii* accession, KU-2078. **(G)** A synthetic hexaploid wheat line with the glaucous phenotype; the line was derived from an interspecific cross between Langdon and a glaucous *Ae. tauschii* accession, KU-2156. **(H)** Geographical distribution of glaucous-type accessions in *Ae. tauschii*. The *Ae. tauschii* accessions were classified into three genealogical lineages, TauL1, TauL2, and TauL3 [19].

Of the 210 *Ae. tauschii* accessions examined, only 20 (9.5%) exhibited the glaucous phenotype and produced whitish wax on the surfaces of peduncles and spikes (Figure 1A-D, Additional file 2). Wax production for each accession was completely consistent between the Fukui and Kobe environments. Each glaucous accession belonged to *Ae. tauschii* subspecies *tauschii*; in other words, none belonged to *Ae. tauschii* subspecies *strangulata*; the geographic distribution of glaucous accessions was limited to the area that spans from Transcaucasia to the southern coastal region of the Caspian Sea (Figure 1H). In the eastern habitats (central Asia, Afghanistan, Pakistan, India, and China) of the species range, no glaucous accession was found. Of the 20 glaucous accessions, 19 belonged to the TauL2 lineage, and only one (IG127015 collected in Armenia) belonged to the TauL1 lineage (Additional file 2).

Of the 82 synthetic wheat lines that we examined, 15 exhibited whitish wax production on the peduncle and spike surface (Figure 1E-G), whereas no wax production was evident in any of the 67 other lines (Additional file 2). Of the 15 lines that showed the glaucous phenotype, 13 were produced by crossing Ldn with glaucous *Ae. tauschii* accessions, and each of the 67 non-glaucous lines was produced by crossing Ldn with a non-glaucous *Ae. tauschii* accession. Notably, two synthetic lines, Ldn/KU-2104 and Ldn/KU-2105, exhibited the glaucous phenotype even though their parental *Ae. tauschii* accessions were non-glaucous.

### Mapping of the *Iw2* locus in *Ae. tauschii* and synthetic wheat

Two F_2_ populations of *Ae. tauschii* and three F_2_ populations from the synthetic wheat lines were analyzed to map the loci that control inhibition of wax production. Each F_1_ plant used for the five cross combinations exhibited the non-glaucous phenotype. In each F_2_ population, the ratio of non-glaucous to glaucous individuals was 3:1; these findings were statistically significant and consistent with Mendelian segregation of alleles of a single gene (Table 1). These results indicated that a single genetic locus was associated with the phenotypic difference between non-glaucous and glaucous surfaces on peduncles and spikes, and that allele conferring the non-glaucous phenotype was dominant and the allele conferring the glaucous phenotype was recessive.

**Table 1:** **Segregation analysis of the non-glaucous phenotype in the five F**_**2**_ **mapping populations**

**F**_**2**_ **population**	***N***	**Non-glaucous type**	**Glaucous type**	**χ** ^**2**^ value*	***P*** **value**
KU-2003/KU-2124	116	89	27	0.184	0.668
KU-2154/KU-2126	116	78	38	3.724	0.054
Ldn/KU-2159//Ldn/IG126387	100	71	29	0.853	0.356
Ldn/KU-2124//Ldn/IG47259	82	65	17	0.797	0.372
Ldn/PI476874//Ldn/KU-2069	106	77	29	0.314	0.575

*Expected segregation ratio was 3:1.

**Figure 2:**
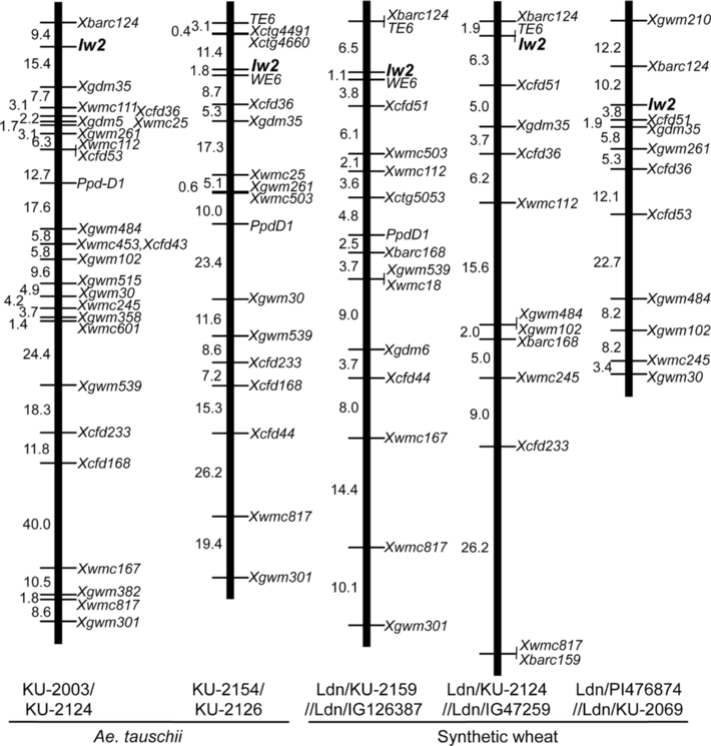
**Linkage maps of** ***Iw2*** **on chromosome 2D.** Two and three mapping populations were generated for *Ae. tauschii* and synthetic hexaploid wheat, respectively. Genetic distances are represented in centimorgans to the left of each chromosome.

A single locus that controlled inhibition of wax production in *Ae. tauschii* was mapped to the same region of the short arm of chromosome 2D in each F_2_ mapping population (Figure 2). In the KU-2003/KU-2124 population, the locus that controlled inhibition of wax production, together with the loci for 25 SSR markers and *Ppd-D1*, was assigned to chromosome 2D, and the map length was 230.0 cM with an average inter-loci interval of 8.85 cM. In the KU-2154/KU-2126 population, the locus that controlled inhibition of wax production, together with 14 SSR and 2 STS markers and *Ppd-D1*, was assigned to chromosome 2D, and the map length was 175.4 cM with average inter-loci spacing of 10.32 cM. In the three synthetic wheat populations, Ldn/KU-2159//Ldn/IG126387, Ldn/KU-2124//Ldn/IG47259, and Ldn/PI476874//Ldn/KU-2069, the locus that controlled inhibition of wax production was mapped to a similar position on the short arm of chromosome 2D (Figure 2). In these three synthetic wheat populations, the locus that controlled inhibition of wax production was mapped together with 11 to 13 SSR markers, 0 to 2 STS markers, and *Ppd-D1*; additionally, the map lengths ranged from 79.4 to 93.8 cM with an average inter-loci spacing of 4.96 to 8.53 cM.

*WE6* and *TE6* are EST-derived STS markers that are linked to *Iw2* in two mapping populations [7,9]. In three of our mapping populations, linkage of the non-glaucousness loci to *WE6* and *TE6* were confirmed. Thus, the position of one locus that controlled inhibition of wax production in *Ae. tauschii* corresponded to the well-known wax inhibitor gene, *Iw2*, on chromosome 2D [6,7]. Therefore, hereafter, all glaucousness-related loci mapped in this study were considered to be identical to *Iw2*.

### Fine mapping of the *Iw2* locus

**Figure 3:**
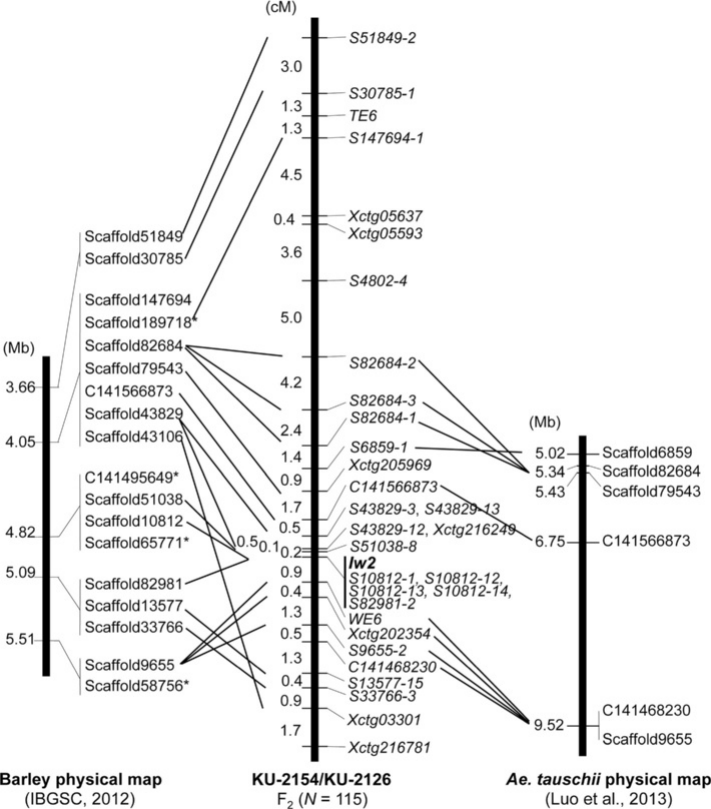
**Comparison of the** ***Iw2*** **linkage map, which contains the** ***Ae. tauschii*** **scaffolds, with the physical maps of barley and** ***Ae. tauschii*** **.** The *Ae. tauschii* scaffolds were assigned to regions of the barley physical map of chromosome 2H [33]. An *Ae. tauschii* physical map with the mapped scaffolds [21] is represented. Scaffold positions (Mb) and numbers [20,21] are shown on the left and right of each chromosome, respectively.

The high-confidence SNPs derived from *Ae. tauschii* RNA-seq data have been plotted onto barley chromosomes [32], and physical map information for the barley genome is available [33]. Additionally, physical map information for *Ae. tauschii* and 16,876 scaffolds that constitute 1.49 Gb from the draft *Ae. tauschii* genome sequence are anchored to the *Ae. tauschii* linkage map [20,21]. The RNA-seq-derived SNP information [31,32] was used to map seven high-confidence SNPs, represented as *Xctg* loci in Figure 3, throughout the *Iw2* chromosomal region in the KU-2154/KU-2126 F_2_ population. Of the seven *Xctg* loci, four were located within the 8.8 cM chromosomal region immediately surrounding *Iw2*. Nucleotide sequences of the four cDNAs corresponding to these *Xctg* loci were used as queries to select the carrier scaffolds from *Ae. tauschii* sequences. We selected the *Ae. tauschii* scaffolds that mapped near the *Xctg*-carrying *Ae. tauschii* scaffolds based on synteny between the wheat and barley genomes and the barley physical map [39]. In all, 18 *Ae. tauschii* scaffolds were assigned *in silico* to an area of the *Ae. tauschii* genome that corresponded to the *Iw2* region in the physical map of barley chromosome 2H (Figure 3). Using a previously developed physical map of the *Ae. tauschii* 2DS chromosome [21], we mapped six *Ae. tauschii* scaffolds *in silico* to the corresponding region in the 2DS physical map. Nucleotide sequences of the selected scaffolds were used to design CAPS or SSR markers for each scaffold, and the markers that were polymorphic between KU-2154 and KU-2126 were then mapped in the F_2_ population (Figure 3).

Of the selected scaffolds, 23 were mapped to the *Iw2* chromosomal region on 2DS, and the remaining three scaffolds were assigned to other chromosomes. In the KU-2154/KU-2126 population with 115 F_2_ individuals, the *Iw2* locus was mapped within the 1.1 cM interval between the most closely linked markers (Figure 3). A dominant marker (S51038-8), derived from the *Ae. tauschii* scaffold 51038 sequence, was located 0.2 cM distal to *Iw2*, and the *WE6* SSR marker was located 0.9 cM proximal to *Iw2*. Five co-dominant markers, derived from two *Ae. tauschii* scaffolds 10812 and 82981, co-localized with *Iw2*. The marker order in the KU-2154/KU-2126 linkage map was generally conserved with that in the barley 2H physical map. However, barley scaffold 9655 was more closely linked to the barley *Iw2* ortholog than were two corresponding *Ae. tauschii* scaffolds, 13577 and 33766, to the *tauschii Iw2* ortholog; this positioning indicated that a local inversion had occurred in the region proximal to *Iw2* during the divergence between barley and *tauschii*.

**Figure 4:**
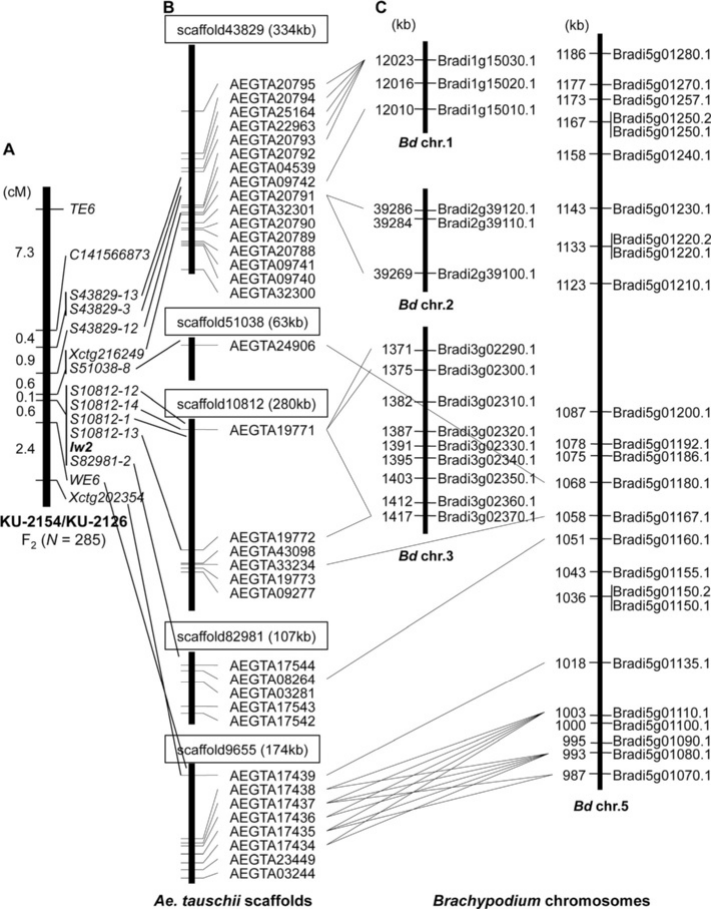
**Assignment of protein-encoding genes found on the scaffolds around** ***Iw2*** **to orthologs on** ***Brachypodium*** **chromosomes. (A)** Linkage map of the region around *Iw2* generated with 285 F_2_ individuals. Genetic distances (cM) are shown on the left, and markers on the right. **(B)** The figure shows the positions of putative genes and mapped markers in the *Ae. tauschii* scaffolds anchored to the *Iw2* region. **(C)** The *Iw2*-orthologous regions on *Brachypodium* chromosomes based on the blastx search of anchored *Ae. tauschii* genes. *Brachypodium* genes are shown on the right, and their position (kb) on the left.

Next, F_2_ individuals of the KU-2154/KU-2126 population and 12 markers from five *Ae. tauschii* scaffolds were used to construct a fine map of *Iw2* (Figure 4A). Based on this linkage map, *Iw2* was located within the 0.7 cM between *Xctg216249*/*S51038-8* and *WE6* and co-localized with five markers derived from two scaffolds, 10812 and 82981. Each of the five scaffolds was 63 to 334 kb in length and included one to 16 putative protein-coding genes [20,21]; marker positions of each scaffold are indicated in Figure 4B. Of the 12 markers, eight were derived from intergenic regions, the other four from open reading frames.

In all, 36 genes were evident on the five scaffolds, and gene annotation could be confirmed for 27 of the 36 genes (Table 2). Of these 27 *Ae. tauschii* genes, 10 putatively encoded cytochrome P450 monooxygenase proteins, and eight encoded disease-related proteins. Additionally, genes encoding laccase, agmatine coumaroyltransferase, receptor kinase, and cell number regulator 2-like were found on the two scaffolds that co-localized with *Iw2*.

**Table 2:** **Colinearity between** ***Ae. tauschii*** **and** ***Brachypodium*** **in the syntenic genomic regions around** ***Iw2***

***Ae. tauschii*** **gene**	***Brachypodium*** **gene**	**Annotation**
AEGTA20795	Bradi1g15030.1	cytochrome p450 85a1
AEGTA20794	Bradi1g15030.1	cytochrome p450 85a1
AEGTA25164	Bradi1g15030.1	cytochrome p450 85a1
AEGTA22963	Bradi1g15030.1	cytochrome p450 85a1
AEGTA20793	Bradi1g15030.1	cytochrome p450 85a1
AEGTA20792		f-box domain containing protein
AEGTA04539		hypothetical protein F775_04539
AEGTA09742	Bradi1g15010.1	probable fructokinase-1-like
AEGTA20791	Bradi2g39120.1	hypothetical protein F775_20791
	Bradi2g39100.1	
AEGTA32301	Bradi3g18920.1	hypothetical protein F775_32301
AEGTA20790		cytochrome p450
AEGTA20789		cytochrome p450 monooxygenase cyp71d70
AEGTA20788		cytochrome p450
AEGTA09741	Bradi2g27777.1	cytochrome p450 71c4
AEGTA09740	Bradi5g01360.1	sulfotransferase 16-like
	Bradi4g37480.1	
	Bradi3g03460.1	
AEGTA32300	Bradi2g10230.2	deleted in split hand split foot protein 1
	Bradi2g10230.1	
AEGTA24906	Bradi5g01180.1	brown planthopper-induced resistance protein 1
AEGTA19771	Bradi3g02290.1	laccase-15-like
	Bradi3g02300.1	
	Bradi3g02370.1	
	Bradi4g11840.1	
AEGTA19772	Bradi4g36820.1	agmatine coumaroyltransferase-2-like
	Bradi3g02310.1	
	Bradi4g36850.1	
AEGTA43098		protein da1-related 1-like
AEGTA33234	Bradi5g01167.1	disease resistance protein rpm1
AEGTA19773		l-type lectin-domain containing receptor kinase -like
AEGTA09277		cytochrome p450 84a1
AEGTA17544	Bradi5g01167.1	disease resistance protein rpm1
AEGTA08264	Bradi5g01160.1	protein da1-related 1-like
AEGTA03281		cell number regulator 2-like
AEGTA17543	Bradi1g30630.1	cell number regulator 2-like
	Bradi3g46930.1	
	Bradi5g12460.1	
AEGTA17542	Bradi1g33650.1	serine threonine-protein kinase receptor
	Bradi1g05890.1	
	Bradi1g75950.1	
	Bradi3g41060.1	
AEGTA17439	Bradi5g01135.1	probable pectate lyase 15-like
AEGTA17438	Bradi5g01110.1	disease resistance rpp13-like protein 1-like
	Bradi5g01080.1	
AEGTA17437	Bradi5g01070.1	disease resistance rpp13-like protein 1-like
	Bradi5g01080.1	
	Bradi5g01110.1	
AEGTA17436	Bradi5g01080.1	disease resistance rpp13-like protein 1-like
	Bradi5g01110.1	
AEGTA17435	Bradi5g01110.1	disease resistance rpp13-like protein 1-like
	Bradi5g01070.1	
	Bradi5g01080.1	
AEGTA17434	Bradi5g01080.1	disease resistance rpp13-like protein 1-like
	Bradi5g01110.1	
AEGTA23449		hypothetical protein F775_23449
AEGTA03244		hypothetical protein F775_03244

The *Ae. tauschii* scaffolds that included protein coding genes were used as queries to search the *Brachypodium* genomic information via a blastn search. Of the *Ae. tauschii* genes on the five scaffolds, 18 had obvious orthologs in the *Brachypodium* genome (Figure 4C). Putative orthologs of the *Ae. tauschii* genes from the four scaffolds were assigned to the 987 to 1068 kb region of *Brachypodium* chromosome 5. In addition, three *Brachypodium* paralogs (Bradi5g01220.1, Bradi5g01220.2, and Bradi5g01230.1) positioned in the 1133 to 1143 kb region were orthologous to an *Ae. tauschii* gene, AEGTA20985; additionally, Bradi5g01280.1 at 1186 kb was orthologous to AEGTA28084 in scaffold 6859. The locations of two *Ae. tauschii* genes, AEGTA20985 and AEGTA28084, were 3 and 3.9 cM, respectively, distal to *Iw2* (Figure 3); therefore, the distal part of *Iw2* showed chromosomal synteny to *Brachypodium* chromosome 5. Thus, the *Iw2* chromosomal region on 2DS was generally syntenic to *Brachypodium* chromosome 5. However, putative orthologs of the *Ae. tauschii* genes from scaffold 43829 were assigned to *Brachypodium* chromosomes 1 and 2. Two paralogous *Ae. tauschii* genes, AEGTA19771 and AEGTA19772, on scaffold 10812 were orthologous to three paralogous *Brachypodium* genes (Bradi3g02290.1, Bradi3g02300.1, and Bradi3g02370.1) on *Brachypodium* chromosome 3. Therefore, the chromosomal synteny between *Ae. tauschii* and *Brachypodium* around the *Iw2* orthologs was complex with regard to chromosome structure.

### *Iw2*-linked marker genotypes in *Ae. tauschii*

To determine the genetic associations among the developed markers and glaucousness, 13 *Iw2*-linked PCR markers—including five CAPSs, five SSRs, one HRM, one insertion/deletion (indel), and one dominant (presence or absence) marker—were used to genotype the 210 *Ae. tauschii* accessions (Table 3). For eight of the 13 markers, the 210 accessions exhibited just two apparent alleles; additionally, the set of accessions exhibited just three distinct electrophoresis patterns—including the KU-2154-type, the KU-2126- type, and one other type—at one SSR marker for *WE6*. The other four SSR markers were highly polymorphic among the accessions; specifically, each marker gave rise to more than three distinct electrophoresis patterns.

**Table 3:** **Association between** ***Iw2*** **-linked marker genotypes and glaucous versus non-glaucous phenotypes in 210 accessions of** ***Ae. tauschii*** **and the distribution of marker genotypes among Iranian wheat landraces**

**Marker name**	**Marker type**	**No. accessions**	**Glaucous phenotype (** ***N*** **= 20)**			**Non-glaucous phenotype (** ***N*** **= 190)**			***P*** **-value for** ***F*** **-test in the association analysis** ^**a**^	**Iranian wheat landraces (** ***N*** **= 17)**
			**KU-2154 -type**	**KU-2126 -type**	**Others**	**KU-2154 -type**	**KU-2126 -type**	**Others**		
*C141566873*	CAPS	210	0	20	0	91	99	0	0.403	KU-2126-type
*S43829-13*	SSR	210	4	5	12	65	22	102	8.20E-05	Other types
*S43829-3*	CAPS	206	17	2	0	184	3	0	-	KU-2154-type
*S43829-12*	SSR	207	0	6	14	136	37	14	1.55E-07	KU-2126-type (15)/Others (2)
*Xctg216249*	HRM	210	9	11	0	177	13	0	1.52E-04	KU-2154-type
*S51038-8*	Dominant	210	0	20	0	170	20	0	8.18E-10	KU-2154-type
*S10812-12*	CAPS	210	18	2	0	170	20	0	1.55E-05	KU-2126-type
*S10812-14*	Indel	197	0	20	0	145	32	0	8.66E-11	KU-2126-type
*S10812-1*	SSR	210	0	15	5*	135	0	55 (4)	3.26E-24	Other types
*S10812-13*	CAPS	206	0	20	0	180	16	0	9.92E-16	KU-2126-type
*S82981-2*	SSR	210	0	15	5*	136	0	54 (5)	1.95E-22	Other types
*WE6*	SSR	210	0	14	6*	59	56	75 (75)	0.169	Other types
*Xctg202354*	CAPS	210	0	20	0	113	77	0	0.041	KU-2126-type

The numbers of accessions for each genotype are represented in glaucous and non-glaucous phenotypes.

The numbers of non-glaucous-type accessions showing the genotype corresponding to the other one in the glaucous-type accessions are indicated in parenthesis.

*These accessions showed the same genotype different from KU-2154 and KU-2126.

^a^The values were calculated based on a mixed linear model in the TASSEL ver. 4.0 software.

The association analysis showed that four SSR markers (*S43829-13*, *S43829-12*, *S10812-1*, and *S82981-2*), an HRM marker (*Xctg216249*), the dominant marker (*S51038-8*), an indel marker (*S10812-14*), and two CAPS markers (*S10812-12*, and *S10812-13*), co-localized with *Iw2* in the *Ae. tauschii* linkage map, were significantly (*P* < 1E-3) associated with variation in glaucousness; in contrast, the other three genotyped markers were not significantly associated with variation in glaucousness (Table 3). The CAPS marker *S43829-3* was removed from this association analysis because of the low-frequency (<0.05) allele. In particular, the KU-2126-type allele of the SSR locus *S10812-1* was found only in 15 of the 20 glaucous accessions; moreover, none of the 190 non-glaucous accessions carried this KU-2126-type allele. The other five glaucous accessions carried a third allele of the *S10812-1* locus. In 55 of the 190 non-glaucous accessions, only four carried the third allele of the *S10812-1* locus, and the other 135 accessions carried different *S10812-1* alleles. Of the four exceptional non-glaucous accessions that carried the third *S10812-1* allele, two were KU-2104 and KU-2105, and these had each been used to generate a synthetic hexaploid wheat line Ldn/KU-2104 and Ldn/KU-2105, respectively; both synthetic lines showed the glaucous phenotype (Additional file 2). However, the phenotype of each synthetic hexaploid line (Ldn/KU-2074 and Ldn/KU-2079) derived from the remaining two of the exceptional accessions (KU-2074 and KU-2079) was non-glaucous. Therefore, phenotypic differentiation in glaucousness was almost completely explained by the allelic configuration at the *S10812-1* locus in these natural populations of *Ae. tauschii*.

The 17 Iranian wheat landraces showed the KU-2154-type alleles at *S43829-3*, *Xctg216249*, and *S51038-8*, whereas they exhibited the KU-2126-type alleles at *C141566873*, *S10812-12*, *S10812-14*, *S10812-13*, and *Xctg202354*. In addition, these landraces exhibited various genotypes that differed from the allelic combinations found in *Ae. tauschii* accessions at *WE6* and four SSR marker loci, *S43829-13*, *S10812-1*, *S82981-2* (Table 3). At *S43829-12*, 15 landraces showed the KU-2126-type genotype, and two exhibited other genotypes.

## Discussion

### Natural variation for wax production in *Ae. tauschii*

Glaucousness is presumably among the components of the domestication syndrome in tetraploid wheat [5,6]. Therefore, glaucousness was apparently a target of artificial selection during tetraploid domestication and common wheat speciation; nevertheless, whether glaucousness is an adaptive trait in wild wheat species remains unclear. Cuticular wax on plant surfaces plays an important role in reducing water loss under drought stress conditions for *Arabidopsis* and rice [1,4], and observations in these other species indicate that relationships between glaucousness and drought stress tolerance are tight. Presence of either the *Iw1* or *Iw2* allele greatly reduces ß-diketones in the wax components of plants, resulting in a non-glaucous phenotype [3,10]. Comparative study of glaucousness-related genes in near-isogenic lines (NILs) of a common wheat cultivar (S-615) (BC_10_F_3_ generation; [6]) demonstrates that *Iw* alleles had a negative impact on drought tolerance [3]. However, another study of *Iw1* in a NIL (BC_2_F_3_ generation) of common wheat did not detect an association between *Iw1* genotype and water-use efficiency [10].

In this study, we used a set of 210 accessions that represented the entire geographical range of *Ae. tauschii* to examine natural variation in wax production among *Ae. tauschii*, and found 20 glaucous accessions that were collected in the area that spans from Transcaucasus to the southern-eastern coastal region of the Caspian Sea (Figure 1, Additional file 2). In a previous study of 176 *Ae. tauschii* accessions collected from 105 different habitats throughout Afghanistan, Pakistan, and Iran, 17 glaucous accessions were found in this same area that spans from Transcaucasus to the southern-eastern coastal region [16]. Therefore, our findings were fully consistent with previous observations.

Most glaucous accessions belonged to the TauL2 lineage (Additional file 2). TauL2 accessions derived from geographically wide-spread sites throughout the Transcaucasus/Middle East region; these sites represented the western habitats of *Ae. tauschii* [19]. TauL1 accessions were collected from sites widely distributed throughout the species range, and most TauL1 accessions showed a non-glaucous phenotype. Notably, one TauL1 accession (IG127015), collected in Armenia, showed a glaucous phenotype, and the collection site was located in the middle of an area where glaucous TauL2 accessions were collected (Figure 1). Genotyping data suggested that IG127015 had an *Iw2* chromosomal region that was very similar to the *Iw2* chromosomal region of the glaucous TauL2 accessions. One possible explanation for this observation is that IG127015 acquired the *Iw2* chromosomal region from some glaucous individual of the TauL2 lineage. Such introgression could occur in the natural habitat where IG127015 was originally sampled and in experimental fields where the accession was propagated for several generations. Another explanation is that IG127015 became a wax producer through a *de novo* recessive mutation at the *Iw2* locus; this scenario, however, is unlikely because the molecular marker genotypes in the *Iw2* chromosomal region of IG127015 were largely identical to those in the *Iw2* chromosomal region of glaucous TauL2 accessions (Table 3).

Whether the glaucous phenotype of the exceptional TauL1 accession was due to introgression of a glaucous allele from a glaucous TauL2 plant may be difficult to discern. Genome-wide marker analyses using SNP array and diversity arrays technology (DArT) systems indicated that TauL2 was clearly distinct and genetically differentiated from TauL1 [18,19]. This high level of differentiation indicates that the two genealogical lineages have been reproductively isolated, and that, under natural conditions, inter-lineage hybridization seems to have occurred only rarely [17,18]. Nevertheless, the presence of a glaucous-type TauL1 accession indicated that the hybridization between TauL1 and TauL2 might have occurred, but the number of hybridizations seems to be quite small. Further detailed study is required to clarify the past occurrence of the TauL1-TauL2 inter-lineage hybridization in *Ae. tauschii.*

### Causal loci for variation in glaucousness among *Ae. tauschii*

Previous studies show that, in *Ae. tauschii*, the causal gene for the glaucous/non-glaucous phenotypic difference is *Iw2*, and that the genotypes of glaucous and non-glaucous accessions were *W2W2iw2iw2* and *W2W2Iw2Iw2*, respectively [5,14]. The molecular markers tightly linked to *Iw2* were very closely associated with glaucous versus non-glaucous phenotypic difference among the 210 accessions of *Ae. tauschii* (Table 3). Thus, the allelic difference at the *Iw2* locus was the main contributor to the phenotypic difference between the glaucous and non-glaucous accessions of *Ae. tauschii* (Figure 2, Table 3). In common wheat, the markers derived from Bradi5g01180 and Bradi5g01160 are tightly linked to *Iw2* as well as *Iw1* [10,11]. Because the loci that control the glaucous versus non-glaucous phenotypic difference in *Ae. tauschii* mapped to the chromosome 2DS region where the common wheat *Iw2* gene resides (Figure 4), the same *Iw2* gene is likely involved in wax production in both *Ae. tauschii* and common wheat. Actually, although most SSR markers around the *Iw2* region were highly polymorphic among the *Ae. tauschii* accessions and Iranian wheat landraces, three markers co-localized with *Iw2* in *Ae. tauschii S10812-12*, *S10812-14*, and *S10812-13*; notably, each showed the KU-2126-type alleles in each of the 17 Iranian wheat landraces (Table 3). These results indicated that the Iranian wheat landraces, which exhibited the glaucous phenotype, had the *iw2iw2* genotype.

Marker order and gene order around *Ae. tauschii Iw2* was well conserved with those on barley chromosome 2HS and *Brachypodium* chromosome 5 (Figures 3 and 4). Similar chromosomal synteny between the *Iw1* region on 2BS and *Brachypodium* chromosome 5 was recently reported based on mapping with common wheat populations [10,11]. In *Ae. tauschii*, scaffold information derived from the draft genome data were available for detailed analysis of chromosomal synteny at the *Iw2* region. Chromosomal order of the selected scaffolds at *Iw2* revealed the occurrence of a local inversion during divergence between barley and *Ae. tauschii* (Figure 3). Moreover, information of predicted genes in the scaffolds showed that putative translocations occurred during divergence between *Brachypodium* and *Ae. tauschii* (Figure 4). These results also indicated that several gaps existed between the *Ae. tauschii* scaffolds. Thus, colinearity among barley, *Brachypodium* and *Ae. tauschii* was observed in the *Iw2* syntenic region, as was reported recently [11], but further screening of *Ae. tauschii* BAC clones may be required for construction of the complete physical map at *Iw2*.

In the genotyping analysis with *Iw2*-linked markers, non-glaucous accessions with the *Iw2Iw2* genotype constituted the majority of all 210 accessions (Table 3). However, four non-glaucous accessions (KU-2074, KU-2079, KU-2104, and KU-2015) shared a genotype at *S10812-1* (the most tightly linked marker) with five glaucous accessions (IG127015, KU-2106, KU-2158, KU-2159, KU-2160), indicating that these four non-glaucous accessions may have the *iw2iw2* genotype in spite of the *S10812-1* genotypes. In fact, synthetic hexaploids from hybrids between Ldn, which has the glaucous genotype (*W1W1iw1iw1*) [10], and two of the four non-glaucous accessions, KU-2104 and KU-2105, exhibited the glaucous phenotype (Additional file 2). In contrast, the phenotypes of all synthetic hexaploids derived from the KU-2074 and KU-2079, were non-glaucous. Accordingly, KU-2074 and KU-2079 seemed to have the *Iw2Iw2* genotype even though they shared an *S10812-1* genotype with the five glaucous accessions. Taken together, all this evidence indicated that *Iw2* was the major gene that controls inhibition of wax production in *Ae. tauschii*.

As yet, no loss-of-function allele has been reported for *W2*, a major wax-producing gene in *Ae. tauschii*. In common wheat, however, some cultivars such as Chinese Spring and Salmon carry the recessive *w2* allele [5]. Similarly, non-glaucous-type accessions with the *w1* recessive allele have been discovered among wild emmer wheat [5]. Whether the recessive loss-of-function mutation occurred at the diploid level (i.e., in *Ae. tauschii*) or at the hexaploid level (i.e., in *T. aestivum*) is not known. Further studies are required to clarify the details of the genetic mechanism that underlies the wax production in *Ae. tauschii*.

### Implication of the *Iw2* variation in hexaploid wheat speciation

Based on a comparative genic analysis among common wheat and its ancestral species, Tsunewaki [5] suggested that common wheat, which is hexaploid, is the product of a hybrid cross that took place between a glaucous cultivated emmer wheat with the genotype *W1W1iw1iw1* and a glaucous wild *Ae. tauschii* with the genotype the *W2W2iw2iw2* genotype in the mountainous region near the southwestern coastal part of the Caspian Sea. Here, we found that, of 210 *Ae. tauschii* accessions, only 20 had the glaucous phenotype (Additional file 2) and that a dominant allele at the *Iw2* locus were responsible for expression of the non-glaucous phenotype (Table 1). Furthermore, we found that, on the basis of the molecular-marker genotypes in the *Iw2* chromosomal region and the phenotypes of the synthetic common wheat lines, virtually all non-glaucous accessions had the *Iw2Iw2* genotype (Table 3, Additional file 2). A non-glaucous accession that had the *iw2iw2* genotype was not found among the 210 accessions. This finding was notable because the double recessive *w2w2iw2iw2* genotype, if present, would have also caused the non-glaucousness phenotype. The reason for the absence of any *Ae. tauschii* accession with the *w2w2 iw2iw2* genotype from this collection was not clear, but this fact may indicate that functional *W2* alleles confer some adaptive advantage under natural conditions. Taken together, the evidence from this study was consistent with the view that glaucous *Ae. tauschii* individuals that had the *W2W2iw2iw2* genotype were involved in the origin of hexaploid common wheat.

Previous evidence based on isozyme variations and DNA marker polymorphisms is consistent with the hypothesis that the birthplace of hexaploid wheat is within a broad area ranging from Armenia to southwestern Caspian Iran [18,40-42]. The geographic range of the parent populations of glaucous *Ae. tauschii* accessions was very consistent with the region postulated in this hypothesis (Figure 1). However, the *Ae. tauschii* subspecies-*strangulata* has been postulated to be the D-genome donor of common wheat [43]. Of the 210 *Ae. tauschii* accessions that we examined, only 12 accessions have markedly moniliform spikes, and each of these were originally collected in the southeastern coastal Caspian region [25,26]. Taxonomically, these accessions could be classified as *Ae. tauschii* Coss. subspecies *strangulata* (Eig) Tzvel. Our data demonstrated that all these *strangulata* accessions, which were not glaucous, had the *Iw2Iw2* genotype (Figure 1). On the assumption that the ancestral *Ae. tauschii* had the *W2W2iw2iw2* genotype, this finding may suggest that the southeastern coastal Caspian populations of *Ae. tauschii* subspecies *strangulata* do not represent the direct descendants of the ancestral populations that gave rise to hexaploid common wheat.

## Conclusions

Analysis of the *Iw2* locus may contribute to improve our understanding of the evolution of hexaploid wheat. Of the 210 *Ae. tauschii* accessions, only 20 glaucous accessions were found in the area that spans from Transcaucasia to the southern coastal region of the Caspian Sea. Of the 82 synthetic wheat lines that we examined, 15 were glaucous, and each of the 67 non-glaucous lines was produced by crossing Ldn with a non-glaucous *Ae. tauschii* accession. Of the 15 glaucous lines, 13 were produced by crossing Ldn with glaucous *Ae. tauschii* accessions. The remained two accessions seemed to have the *Iw2Iw2* genotype according to the genotyping analysis with the *Iw2*-linked markers. Therefore, allelic differences at the *Iw2* locus on the short arm of chromosome 2D were the main contributors to the phenotypic difference between the glaucous and non-glaucous accessions of *Ae. tauschii*. Some molecular markers, such as *S10812-1*, closely linked to *Iw2* were significantly associated with variation in glaucousness in *Ae. tauschii*. These results suggest that the D-genome donor of common wheat could have been any *Ae. tauschii* variant that carried the recessive *iw2* allele.

### Availability of supporting data

The data sets supporting the results of this article are included within the article and its supplementary files.

## Additional files

Additional file 1
**List of primers developed in this study.**
Additional file 2
**List of all 20** ***Ae. tauschii*** **glaucous accessions and wheat synthetics with the glaucousness phenotype in the 82 synthetic hexaploid wheat lines.**

## Abbreviations

CAPS: Cleaved amplified polymorphic sequence
HRM: High resolution melting
Ldn: Langdon
SNP: Single nucleotide polymorphism
SSR: Simple sequence repeat
