# Supplementary material for: The cuticular wax inhibitor locus Iw2 in wild diploid wheat Aegilops tauschii: phenotypic survey, genetic analysis, and implications for the evolution of common wheat
Source: BMC Plant Biol. 2014 Sep 16;14:246. doi: 10.1186/s12870-014-0246-y (PMC4172845; doi:10.1186/s12870-014-0246-y)
Supplement: Additional file 1 — List of primers developed in this study. [file 12870_2014_246_MOESM1_ESM.pdf]

Additional file 1. List of primers developed in this study

Maker name	Primer sequences (5'- to -3')	Type	Restriction enzyme	Annealing temp. (°C)
S51849-2	CTCGTCGAAATCTGCCTCGA<br>ATCGAGTCGGGAATCAGCAC	Dominant	–	58
S147694-1	AGTATTGACAGTACAAGACCCGT<br>CCCCCTTCAACTGACATAAGCA	SSR	–	58
S30785-1	AAAAGAAGAAAACCACGAAATAAACA<br>ACCTGCAACTAGTGGCTTTT	SSR	–	58
S4802-4	CACTCATCACGGCAGTCACT<br>ACAACAAGCAATGTGAGGCC	SSR	–	58
S82684-2	ACACCCACACCACACGTAT<br>GACTTCGACCACGTCCAAGG	CAPS	*Acc*I	58
S82684-3	ACAATACCGGGAGTCCTCGC<br>TGCCCATGAATAGAGAAAGTTTGT	CAPS	*Hha*I	58
S82684-1	TCTTTCTTTGCGGGGACACT<br>CAAGGTGATCCTGACTGGCC	Dominant	–	58
S6859-1	AGTGTTCCTACTGTTCAATAGTGT<br>AAGCTGGTTGTAGTGCACCT	SSR	–	58
Xctg205969	CCTTTCTTTGGCGACCAACC<br>AGCATCAACCAACTTCTGCA	HRM	–	60
C141566873	TGGAGCCCACAATAAAGAAAGC<br>ACAATTTGCATTTATGGCAGAGCT	CAPS	*Nco*I	58
S43829-13	ACCCAAGATAGAACCACCCG<br>GAGCAGAGTACAGACAGCCG	SSR	–	58
S43829-3	GCGTGCAGGCAGAGTTTTTA<br>TTTTGGGTTGACCTGGCGAA	CAPS	*Hha*I	58
S43829-12	GCCAAGGAGCAAGGAAAAGC<br>TGGGCTTGGGATATGCTCTC	SSR	–	58
S51038-8	TGGCAGTGAAACCTCCCAAA<br>AAACATCACCACATTTCTCTAGTTTG	Dominant	–	54
S10812-12	TCTATACCTTTGCATTGGCGA<br>CGCTACTGATAGGCCAAAAACC	CAPS	*Hha*I	58
S10812-14	ATTTCGAGCATCTCTAGCTTCA<br>GTTGCTGTCTTTGACGGTCC	Indel	–	58
S10812-1	TACAACCTTGGGCCAGTCAC<br>CGTCGTCTTCCCTACAAATCG	SSR	–	58
S10812-13	CTCTTCAAGCCCCGATCCG<br>ACGGAGAAGTGTTATCCAGTCC	CAPS	*Hha*I	58

S82981-2	TGTACACGTCACTTCAACGC<br>AGTCAGCGATCACGTATGCA	SSR	–	58
Xctg202354	CATTGCCTTGTCCTTGAGGT<br>AAGAATTGTCGGTTGGCTGT	CAPS	*Hae*III	58
C141468230	ACAAGTGCACAATTTACCAGCA<br>CCACCAGGTCTTCTCTGCAC	HRM	–	60
S9655-2	TCAACATTGCCATCCATGCG<br>TGGTGTGTGATCTGGGCAAA	CAPS	*Kpn*I	58
S13577-15	GGTGGAGAGGTTACAGCACC<br>TGTGTGTGTCAGCATTACGGA	CAPS	*Eco*T22I	58
S33766-3	CTCGCGGTCTAGGGTTGGC<br>GCGCACCCCGAAACAATTTA	CAPS	*Hae*III	58
Xctg03301	GCCATATTGGTCGAAATTTCGGT<br>GTACCCCTGCACATCCAGAT	CAPS	*Hha*I	58
