# Supplementary material for: The cuticular wax inhibitor locus Iw2 in wild diploid wheat Aegilops tauschii: phenotypic survey, genetic analysis, and implications for the evolution of common wheat
Source: BMC Plant Biol. 2014 Sep 16;14:246. doi: 10.1186/s12870-014-0246-y (PMC4172845; doi:10.1186/s12870-014-0246-y)
Supplement: Additional file 2 — List of all 20 Ae. tauschii glaucous accessions and wheat synthetics with the glaucousness phenotype in the 82 synthetic hexaploid wheat lines. [file 12870_2014_246_MOESM2_ESM.pdf]

Additional file 2. List of all 20 *Ae. tauschii* glaucous accessions and wheat synthetics with the glaucousness phenotype in the 82 synthetic hexaploid wheat lines

Accession number	Collected country	PCA group[b]	Synthetic wheat with Ldn
KU-2811	Armenia	TauL2	glaucous
IG127015	Armenia	TauL1	n.d.
AE1037	Georgia	TauL2	n.d.
KU-2827[a]	Georgia	TauL2	n.d.
KU-2069	Iran	TauL2	glaucous*
KU-20-7	Iran	TauL2	n.d.
KU-20-8	Iran	TauL2	glaucous
KU-2086	Iran	TauL2	n.d.
KU-2106	Iran	TauL2	glaucous*
KU-2111	Iran	TauL2	glaucous
KU-2112	Iran	TauL2	n.d.
KU-2118	Iran	TauL2	glaucous
KU-2124	Iran	TauL2	glaucous
KU-2126	Iran	TauL2	glaucous
KU-2155	Iran	TauL2	glaucous
KU-2156	Iran	TauL2	glaucous*
KU-2158[c]	Iran	TauL2	glaucous*
KU-2159	Iran	TauL2	glaucous
KU-2160[c]	Iran	TauL2	glaucous
PI486267	Turkey	TauL2	n.d.
KU-2104[d]	Iran	TauL2	glaucous
KU-2105[d]	Iran	TauL2	glaucous*

[a]Glaucousness was not necessarily observed in all growth seasons.
[b]Matsuoka et al. [19]
[c]variety *meyeri*
[d]The parental *Ae. tauschii* accessions were non-glaucous although their derived synthetic wheat lines were glaucous.
*Glaucousness was confirmed in two synthetic hexaploid lines independently twice produced.
n.d., not determined
